# Exploring how IP marketing (media marketing) influences consumer shopping psychology through quantitative and empirical analysis

**DOI:** 10.3389/fpsyg.2024.1292636

**Published:** 2024-01-15

**Authors:** Hang Zhang, Xiaohuan Luo

**Affiliations:** The Department of Marketing Communication at the School of Journalism and Communication, Hangzhou City University, Hangzhou, China

**Keywords:** intellectual property marketing, media marketing, AISAS model, digital marketing, SPSS, AMOS

## Abstract

As the concepts of intellectual property and media marketing have gained popularity, media marketing has gradually become an integral part of intellectual property marketing, and its use has become more widespread. However, the field of intellectual property marketing (media marketing) has become confused and faces challenges such as loss of uniqueness and weak consumer connections. Existing research efforts have focused on marketing strategies for branded intellectual property, but have neglected the important perspective of consumer psychology and behaviour. In this study, we use the AISAS model to segment digital marketing and delve into consumer psychology and behavioural factors that influence intellectual property marketing (media marketing). This exploration covers intellectual property content at the attention stage, intellectual property value at the interest stage, emotional trust at the search stage, mental consumption at the purchase stage and fan interaction at the sharing stage. We conducted a comprehensive analysis of the data using SPSS and AMOS, and integrated a consumer attitude questionnaire. The final findings confirm that intellectual property content, value, emotional trust, spiritual consumption and fan interaction all positively influence consumer psychology and behaviour. In addition, we make consumer-centric recommendations to extend the life cycle of intellectual property and promote sustainable brand development.

## 1 Introduction

Intellectual Property commonly referred to as IP. In the past, it refers to the legal rights generated from the intellectual labor produced by humans. These creations can encompass various forms of creative products, such as innovations, literature, artistic works, trademarks, patents, industrial designs, and more. IP grants creators and innovators exclusive rights to their creations, inventions, or designs, encouraging innovation and safeguarding their economic interests.

The primary forms of intellectual property include the following:

1. Copyright (Reyman, 2009): Used to protect literary and artistic works, such as books, music, films, software, and more. Copyright grants creators the rights to reproduce, distribute, display, and create derivative works from their creations.
2. Trademark (Stim, 2022): Used to identify the source of goods or services, such as brand names, logos, trademark designs, and more. Trademarks protect the unique identifiers of brands, preventing others from using similar marks in related fields.
3. Patent (Lanjouw et al., 1998): Used to protect new inventions and innovations. Patents grant inventors exclusive rights to their inventions for a certain period, preventing others from making, selling, or using similar inventions during the patent’s term.
4. Industrial Design (Brem et al., 2017): Used to protect the visual design of products in industrial and artisanal manufacturing fields, including product shapes, aesthetics, and more. Industrial designs safeguard the appearance features of products, giving them unique characteristics in the market.
5. Geographical Indication (Bramley, 2011): Used to identify goods produced in specific geographical regions with particular qualities, reputation, or other characteristics. Geographical indications protect region-specific agricultural products, handicrafts, and more.

In the past, the protection and management of IP have been crucial for fostering innovation, driving economic development, and safeguarding the rights of creators and innovators. Different countries and regions have established relevant laws and systems to regulate the application, protection, and resolution of disputes related to IP. However, due to the rapid development of the internet and the emergence of many new phenomena (Weiser, 2003), IP has found increased integration and application in various related domains (Drahos, 2016; Bently et al., 2022), leading to mutually reinforcing effects, especially in the field of media marketing. Currently, IP marketing has become a focal point in the field of brand marketing (Robson et al., 2020). After years of development and integration in the domain of brand marketing, IP is no longer confined to the realm of IP alone but has evolved into a phenomenon-level marketing concept. In recent years, media marketing has garnered increasing attention within brands and has been favored by consumers, yielding additional premium effects such as personalization, a sense of value, communication depth, and reputation, naturally channeling consumers’ emotions towards the synergy between the brand and IP. The IP marketing discussed in the following sections of this article primarily pertains to media marketing.

We have primarily reviewed the progress of content marketing, IP marketing, and consumer psychology and behavior. However, there is currently no specific research that elucidates how IP marketing influences consumer psychology and behavior and shopping psychology. In this era of new retail, consumers are at the core of brands. By delving into the factors that impact consumer psychology and behavior, we can better distill effective brand marketing experiences and provide theoretical support for achieving sustainable brand development.

In the realm of content marketing (Ho et al., 2020), content creation needs to align with brand values and value creation in order to craft and disseminate impactful and compelling content. IP marketing (Wang and Zhai, 2021) is an integral component of content marketing. In contrast to the external representation of the brand, brand IP is more likely to evoke a deep sense of individuality and spirit, transcending the mere association with the product itself to extend the business value of the brand. However, the specific quantification of these influences for further analysis of experimental phenomena remains a focal point of our study.

AISAS model (Abdurrahim et al., 2019) has been introduced in the literature, encompassing variables like attention, interaction, search, action, and sharing. The AISAS model aptly quantifies experimental outcomes and derives corresponding conclusions. It indicates that content on social media has a significant impact on people’s attention, interest, and desire for further promotional information, with an individual’s attention and interest markedly influencing decision-making and content sharing. Drawing inspiration from this literature, we aim to delve more deeply into the application of the AISAS model to quantitatively analyze the impact of IP marketing on consumer psychology and behavior. Through unveiling critical factors that enterprises should be attentive to during IP marketing campaigns, we seek to further refine content marketing practices and foster the continuous evolutionary development of brands.

## 2 Literature review

### 2.1 Content marketing

Content marketing is a strategy that attracts customers by creating relevant content (text, videos, eBooks, social media posts). It is a marketing method that does not directly brand, but rather engages the target audience by providing solutions to problems. Lou et al. (2019) suggests that the use of branded content marketing can facilitate consumers to gain value through their social learning process, which in turn can drive beneficial brand evaluation and purchase intentions. The research shows that brands can satisfy the emotional needs of users by producing easy-to-understand and interesting brand content to promote the value of consumers. The limitation of the study is that the selected case content is one-sided, and there is indeed a high correlation between brand loyalty and purchase intention, which may exaggerate the strength of their relationship in the model test.

Ho et al. (2020) propose that the practice of content marketing is a necessity for enterprises seeking to modernize their marketing practices and enhance their online brands through digitalization. The findings point out that content creation needs to be aligned with brand values and value creation, to create and socialize compelling content to have a positive impact. The content that a brand creates and markets for its audience can generate value when consumed and shared. But a limitation of the study is that it does not extend to how to target content marketing to consumers.

Based on the literature review of content marketing, we will deeply study IP marketing under content marketing, and establish a relationship with consumers for research.

### 2.2 IP marketing

IP was originally the abbreviation of the English “Intellectual Property,” and the concept of “IP” in the Internet industry has been extended, in addition to literature, animation, film and television, games, it can also be a character, a phenomenon, a hot spot, or a sentence, as long as it can only rely on its own attraction. A cultural asset that breaks free of the shackles of a single platform, gets traffic on multiple platforms, and distributes it, that is an IP. IP specifically refers to the cross-media content management with long-term vitality and commercial value.

IP marketing (Harrer and Lackner, 2014; Mou, 2020; Chen et al., 2023; Sun et al., 2023) refers to the diversified creation of IP content by enterprises on different media, platforms and channels, obtaining traffic by enriching and using the value of their content, and establishing emotional connections with consumers, to promote circle breaking. Luby and Bedford (2015) proposed that a prerequisite for IP marketing is the continuous production of high-quality content for the IP. The creation of high-quality content should consider factors such as guiding customers to consume cultural identity, shaping distinctive and appealing IP, and establishing trust. The literature puts forward the considerations of IP marketing content production, but there is no empirical analysis to explore its actual impact on consumers. In addition, Wang and Zhai (2021) proposed that under the powerful role of IP, it will bring considerable traffic and profits to brands, and at the same time provide consumers with a lifestyle and emotional expression that can be implanted with emotional sustenance. Her research holds that the Disney brand is integrated into the popular era through the operation of IP, essentially improving the connection between people and people or between people and brands through the influence of cultural identity. The research shows that consumers’ emotional attitude toward people and brands is an important purpose of IP marketing. IP marketing has the advantages that brand independent marketing does not have. IP marketing has sustainability, extensibility and cross-platform. If the audience has always been full of interest and emotional identification with an IP, the brand marketed by this IP will always be remembered by the audience, and it will be known by more and more circles due to the circle breaking effect. On the other hand, brand independent marketing activities are timeliness, will not have a long-term impact, it is difficult to form brand impression, and the cost is much higher than IP marketing. The main difference between brand independent marketing and IP marketing is their focus and strategic approach. The former focuses on using a company’s own resources and capabilities to promote its products or services, such as a small business promoting its own handicrafts through social media. In contrast, the latter leverages existing, popular intellectual property (e.g., films, comic book characters, or artwork) to enhance brand appeal, such as a fashion brand launching a clothing line featuring a film character. In short, brand independent marketing focuses more on internal resources and personalized promotion of the brand, whereas IP marketing leverages the appeal of well-known external IP to drive market attention.

However, Ren et al. (2021) believe that there are hidden dangers and confusion in the IP industry co-brand market in the all-media era where new media technology is the mainstream information transmission channel. In the process of rapid development, the compatibility of co-branded products, frequent co-branded products make consumers produce aesthetic fatigue, and the quality and price of co-branded products are uneven. It not only failed to achieve the expected benefits, but also caused certain damage to the image of the joint parties. Therefore, it is necessary to study the factors that influence consumer purchase intention in IP marketing.

### 2.3 AISAS model

American advertising scientist E. S. Lewis proposed the AIDMA model, which theorizes that consumers will go through these 5 stages from contact with information to final purchase: Attention-Interest-Desire-Memory-Action. With the change of communication environment, Dentsu Corporation of Japan (2005) proposed a new consumer psychology and behavior analysis model – AISAS model based on this model, aiming at the change of consumers’ lifestyle in the era of Internet and wireless applications. AISAS model includes: Attention- Interest- Search -Action-Share. It’s a marketing model with network characteristics and a development of AIDMA model.

## 3 Theoretical model and hypothesis

**Figure 1:**
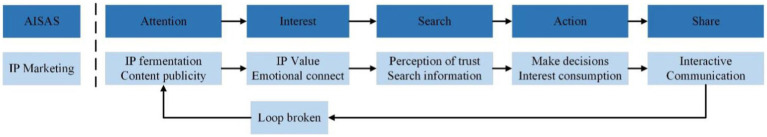
AISAS theoretical model.

AISAS model package has five phases: Attention, Interest, Search, Action and Share, as shown in Figure 1.

**Figure 2:**
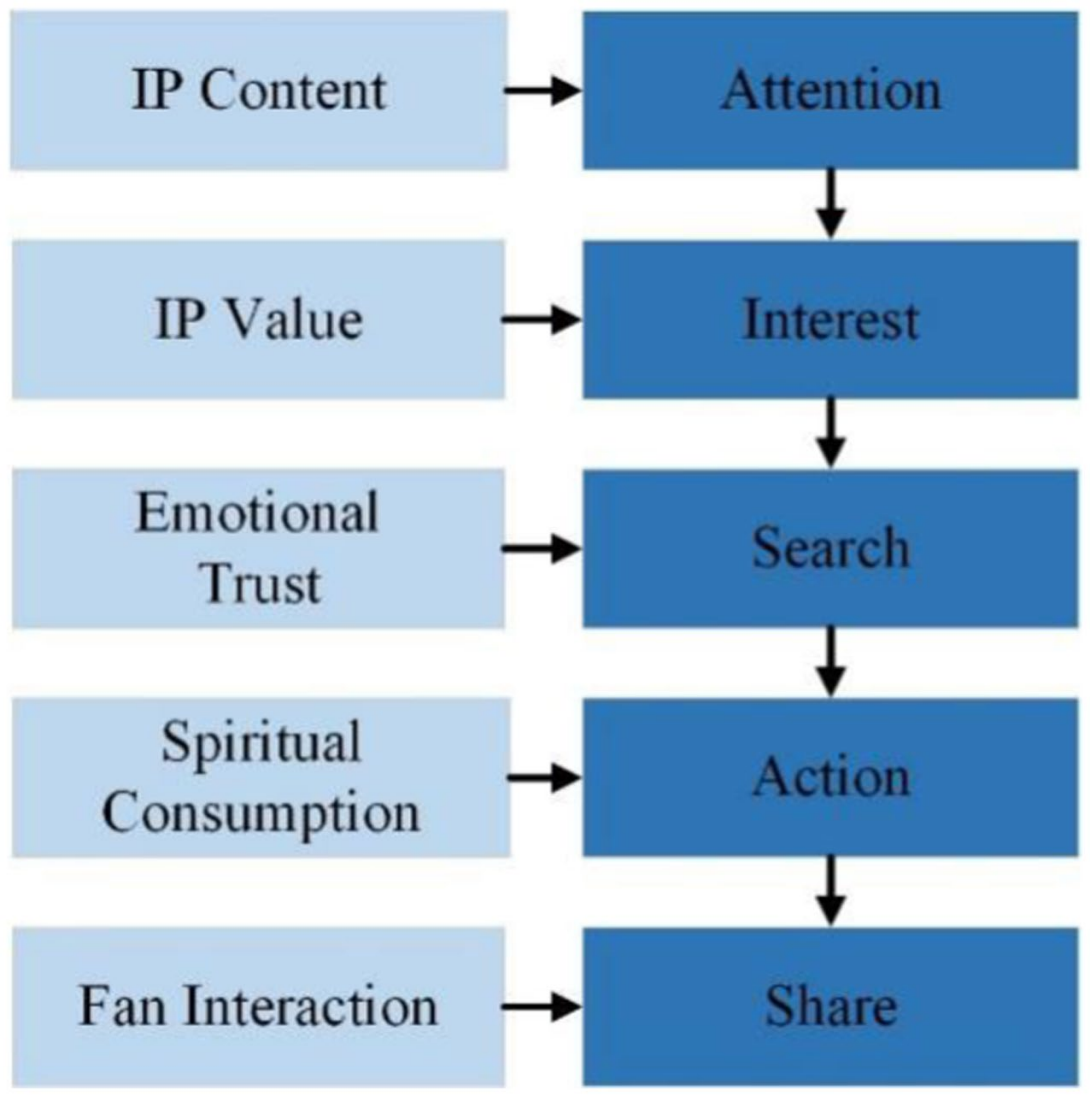
Consumer behavior model in IP marketing based.

This paper builds a relationship model between cultural identity and consumer psychology and behavior based on AISAS model, as shown in Figure 2. In the attention stage, IP image and high-quality content is the core of IP marketing to get attention, this is an era of content is king, interesting, vivid, fresh IP image or infectious, with distinct values of IP content. User interaction and multi-channel, multi-platform, all-weather centralized, blowout behavior to ferment and publicize IP content production make consumers have IP identity, so as to obtain users’ attention. In the interest stage, what kind of value IP can bring to consumers, what kind of needs can meet consumers, and IP that can make consumers identify with will arouse their interest. In the search and action stage, the emotional trust of the brand generated by consumers through IP and the spiritual consumption generated for IP will make the next step after the consumer’s brand recognition. After users have cultural identification with IP and brand, they will make corresponding purchase and sharing behaviors, expand the influence circle through sharing, and even achieve the breakthrough circle to produce explosive funds.

### 3.1 IP content

IP content refers to the content that includes IP image, story plot, cultural thoughts, values, etc. transmitted through multiple platforms and multiple scenes. Zhu et al. (2023) proposed that brands not only rely on rich stories, texts and other content, but also rely on an image IP as a source to attract consumers and occupy the consumer market. Yin and Zhang (2021) expresses the same viewpoint, stating that animation IPs with good storytelling content are more capable of attracting audiences. Ip and Chark (2023) believes that menus have now become a form of IP for restaurants, and anything on the menu that attracts visual attention could be key to the restaurant’s marketing success. Coincidentally, Wan et al. (2023) also proposed that the content of IP has become key to attracting consumers. Based on this, the following hypothesis is proposed:

*H1*: IP content has a positive impact on attention behavior.

### 3.2 IP value

The value of IP to consumers includes cultural value and emotional value. Cultural value includes the content cultural information to be conveyed by IP and the value of content reproduction, whether IP can become a high-quality content source, whether it can interact with users, and whether it can be re-created around and jointly. IP emotional value refers to whether IP has emotional setting, whether it can provide emotional sustenance for consumers, and whether it has the content productivity to softly tell brand stories. Racine (2021) argue that for a brand (IP) to truly meet the needs of customers, it is essential to delve deeply into the cultural value that the brand can create. Simultaneously, it is necessary to establish emotional bonds with users to achieve emotional identification and value alignment with users. Coincidentally, Gong et al. (2022) pointed out that by disseminating high-quality marketing content, enterprises assist in enhancing the depth and quality of content in short video accounts (referred to as brands here). As a result, consumers develop greater interest in the products recommended by the account, a desire to learn more about them, and a higher level of trust. There are many articles in the past that have proposed similar viewpoints (Shen et al., 2021; Bai et al., 2023). In conclusion, the dissemination of high-quality content brings cultural value to consumers, and the satisfaction of curiosity brings emotional value to consumers. Based on this, the following hypothesis is proposed:

*H2*: IP value has a positive effect on interest behavior.

### 3.3 Emotional trust

Trust is the basis of Commodity Exchange between buyers and sellers, and consumers’ trust in IP will directly affect consumers’ purchase behavior. Wongsunopparat and Deng (2021) indicates that when people have sufficient trust in livestreaming hosts engaged in product promotion, it significantly stimulates their desire to make online purchases from these hosts. Coincidentally, the research report by Wang et al. (2022) explored the influence of Chinese internet influencers on consumer attitudes within the context of e-commerce livestreaming. In their study, internet influencers can be regarded as individual Intellectual Properties (IPs), where the influencers’ professional knowledge, bargaining ability, after-sales service, and livestreaming duration all substantially impact consumer trust in them. Not only that, he points out that live shopping is a virtual form of shopping reliant on the internet, and one of the significant ways to facilitate the success of virtual shopping is to nurture a widely trusted online influencers for live sales or product endorsements. When these influencers’ professional knowledge, bargaining ability, after-sales service, and livestreaming duration are all substantially guaranteed, consumers are highly likely to place sufficient trust in them. This, in turn, becomes a critical driving force for generating interest in the product, initiating information-seeking behavior about the product and related items, and ultimately making purchase decisions. In conclusion, since IP marketing aims to enable consumers to contact the brand so as to generate the final purchase behavior, gaining consumer trust through IP is an important driving force to promote consumers’ search behavior for brand information and then make purchase decisions. Accordingly, the following hypothesis is proposed:

*H3*: Emotional trust has a positive effect on search behavior.

### 3.4 Spiritual consumption

Some studies (Park and Namkung, 2022) believe that in IP marketing, consumers use consumption to express their identity, taste, values and other spiritual needs have occupied the main purchase motivation. The audience of IP marketing is mainly young people, whose consumption characteristics are “self-pleasing.” Young people increasingly rely on the “psychological empowerment” and “life reconstruction” effects provided by spiritual activities, and are more willing to pay for their favorite things. Zhang and Phakdeephirot (2023) also indicates that young people are willing to purchase the more expensive and unpredictable Pop Mart blind boxes to satisfy their psychological needs for self-fulfillment. Additionally, Pourazad et al. (2019) revealed the synergistic effect of cognitive (brand attribute association) and affective (E-CBR) factors on luxury brand extension, with brand attribute association and emotional consumer-brand relationship (E-CBR) serving as drivers of brand extension purchase intention. Based on this, the following hypothesis is proposed:

*H4*: Spiritual consumption has a positive effect on action behavior.

### 3.5 Fan interaction

There are four kinds of fan interaction in IP marketing: social media interaction, activity interaction, personalized interaction, and creating community. Social media interactions build bridges of interaction and communication by commenting, liking, sharing and posting relevant content. Engage fans by organizing various online and offline activities. Personalized interactions make fans feel valued and cared for by creating a one-on-one connection with them. Creating community make fans feel a sense of belonging and involvement by building communities, creating social media groups and more. Lin (2023) proposed that in terms of promotion, brands like Miniso adopt an IP marketing strategy of vigorously developing offline immersive interactions to engage fans and customers. This approach will encourage fans to develop cultural identification with the IP and provide opportunities for them to participate in IP marketing, interact, and share. Similarly, Chai (2023) proposed that Disney’s IP marketing strategy involves creating a theme park-like experience to provide fans with a fairytale-like immersive experience. This approach allows fans to develop cultural identification with the IP and drives the sales of IP-related merchandize. Also, Jiang et al. (2022) shows that in sports marketing, cultural identity behavior, experience communication behavior and community maintenance behavior have significant positive effects on consumers’ willingness to co-create value. Based on this, the following hypothesis is proposed:

*H5*: Fan interaction has a positive impact on sharing behavior.

## 4 Empirical research

### 4.1 Questionnaire design and data collection

This study employed an anonymous questionnaire survey method, collecting a total of 214 valid questionnaires. The sample covered respondents of diverse ages, genders, and occupations (detailed in Table 1) to ensure the representativeness and breadth of the data. The selection of the sample aimed to encompass a wide range of consumers to better understand the impact of Intellectual Property (IP) marketing on different consumer behaviors.

**Table 1:** Sample characteristics.

Category		Number of people	Percent
Sex	Man	78	36.45%
	Female	136	63.55%
Age	Under 18 years of age	7	3.27%
	18–25	144	67.29%
	26–35	54	25.23%
	Over 35 years old	9	4.21%
Educational background	Elementary school	5	2.34%
	Junior high school	7	3.27%
	High school/technical school/vocational high school	12	5.61%
	Associate’s degree	28	13.08%
	Bachelor’s degree	142	66.36%
	Master’s degree	17	7.94%
	Doctoral degree	3	1.4%
Average monthly income/living expenses	Less than 1,000	14	6.54%
	1,001–1,500	48	22.43%
	1,501–2,500	55	25.7%
	2,501–5,000	53	24.77%
	5,001–7,500	20	9.35%
	More than 7,500	24	11.21%

The symbol “/” denotes division operation.

The questionnaire data was collected through a combination of online and offline methods. Online, we distributed links to the questionnaire and QR codes through social media and email. Offline participation mainly targeted student groups, relatives, and friends. All questionnaires were filled out anonymously, ensuring the protection of participants’ personal privacy. During the data collection process, we strictly adhered to the ethical standards of the Declaration of Helsinki, ensuring voluntary participation and understanding of the survey’s purpose by the participants.

The questionnaire consisted of three parts: an introduction, basic information collection, and the main body. The main body employed a Likert 5-point scale to measure the mechanisms of IP marketing’s impact on consumer behavior. The scale included multiple dimensions, such as consumer attitudes, behavioral intentions, etc., and its design referenced previous studies in the field (author, year). We particularly considered the applicability of the scale in the Chinese context and made corresponding cultural adaptability adjustments. To ensure the reliability and validity of the scale, we conducted Cronbach’s alpha, KMO, and Bartlett’s sphericity tests, which showed that the scale has good internal consistency and structural validity.

### 4.2 Descriptive statistical analysis

This study’s sample consists of 214 respondents, exhibiting diversity in terms of gender, age, educational background, and monthly income. Regarding gender, female respondents constitute 63.55% of the sample (136 individuals), outnumbering male respondents, who make up 36.45% (78 individuals). This indicates a higher level of participation among women in this survey. In terms of age distribution, the majority of the respondents fall within the 18–25 age group, accounting for 67.29% of the sample (144 individuals), reflecting the dominant position of young consumers in IP consumption. Following this, the 26–35 age group comprises 25.23% of the sample (54 individuals), while respondents under 18 and over 35 years old represent only 3.27 and 4.21%, respectively.

In terms of educational background, a significant majority of the respondents (75.7%) have a bachelor’s degree or higher. Specifically, bachelor’s degree holders constitute 66.36% of the sample, followed by master’s degree holders at 7.94% and doctoral degree holders at 1.4%. This suggests that most participants have a high level of education, which helps them better understand and respond to the questionnaire. Furthermore, the respondents’ monthly income and living expenses indicate a certain level of economic strength, with 71.03% reporting an income exceeding 1,500 yuan, demonstrating their substantial purchasing power.

In summary, the sample of this study shows diversity in gender, age, educational level, and economic capacity, which helps in gaining a deeper understanding of different groups’ attitudes and responses towards IP marketing.

### 4.3 Reliability and validity test

In this paper, we used SPSS 22.0 software to assess the validity and reliability of the data collected and subsequent analyses. The standardized loading coefficients of all variable terms were greater than 0.6, indicating that the observed variables explained the latent variables well. In addition, the Cronbach’s alpha coefficients of all variables were more than 0.6, showing a strong association between the observed indicators and the latent variables. The Cronbach’s alpha coefficient for the overall questionnaire was 0.920, indicating excellent reliability and consistency.

To fully assess the fit of structural equation modelling (SEM), we also calculated model fit indices, including the chi-square/degrees of freedom ratio, root mean square residuals (RMSEA), comparative fit index (CFI) and incremental fit index (IFI). We followed common acceptance criteria, such as RMSEA less than 0.08 and CFI and IFI greater than 0.9, to ensure effective model fit.

In addition, we conducted factor analyses to further validate the validity of the model. The specific experimental results are shown in Table 2. The results of the loadings and correlation analyses of the factors showed that the model was able to explain the associations between the variables well. We paid special attention to the amount of total variance explained in the model to ensure the validity of the factor analysis.

The scales used in this study were validated for adaptation to the Chinese context. This is crucial to scientifically validate the results of a study because only scales validated in a specific context can ensure the accuracy and general applicability of the results.

**Table 2:** Reliability test results.

Variable	Test items	Standardized loadings (Std. estimate)	Cronbach’s α	Composite reliability (CR)	AVE
IP content (GZ)	GZ1	0.802	0.795	0.801	0.574
	GZ2	0.701			
	GZ3	0.767			
IP value (XQ)	XQ1	0.753	0.697	0.700	0.539
	XQ2	0.715			
Emotional trust (SX)	SX1	0.790	0.782	0.783	0.643
	SX2	0.813			
Spiritual consumption (XD)	XD1	0.691	0.711	0.715	0.558
	XD2	0.799			
Fan interaction (FX)	FX1	0.811	0.745	0.749	0.599
	FX2	0.735			

CR, measures internal consistency of latent variables (higher values indicate reliability); AVE, reflects convergent validity, should be >0.5 for good validity.

Validity research is employed to assess the rationality and significance of the research items. Factor analysis serves as the data analysis method for this validation study. Through various indicators such as KMO, commonality, variance explained, and factor loading coefficients, the level of data validity is verified. The survey’s KMO value stands at 0.942, exceeding the threshold of 0.8, signifying that the research data is highly suitable for extracting meaningful information (Table 3).

**Table 3:** Arguments KMO and Bartlett tests.

KMO		0.942
Bartlett test	Approximate chi square	1225.344
	Degree of freedom	55
	Salience	0.000

As shown in Table 4, the commonality value of all the research items exceeded 0.4, which means that information was effectively extracted from these research items. The variance interpretation rate by the factors before and after rotation is 55.742% which is more than the critical value of 50%. This indicates that information was successfully extracted from the research projects.

**Table 4:** Validity test.

Item	Factor load coefficient	Common degree
GZ1	0.792	0.628
GZ2	0.726	0.527
GZ3	0.771	0.594
XQ1	0.781	0.610
XQ2	0.743	0.553
SX1	0.736	0.541
SX2	0.746	0.557
XD1	0.658	0.433
XD2	0.760	0.577
FX1	0.775	0.600
FX2	0.715	0.512
Variance interpretation rate (Before rotation)	55.742%	-
Variance interpretation rate (After rotation)	55.742%	-

### 4.4 Empirical analysis based on structural equation model

**Figure 3:**
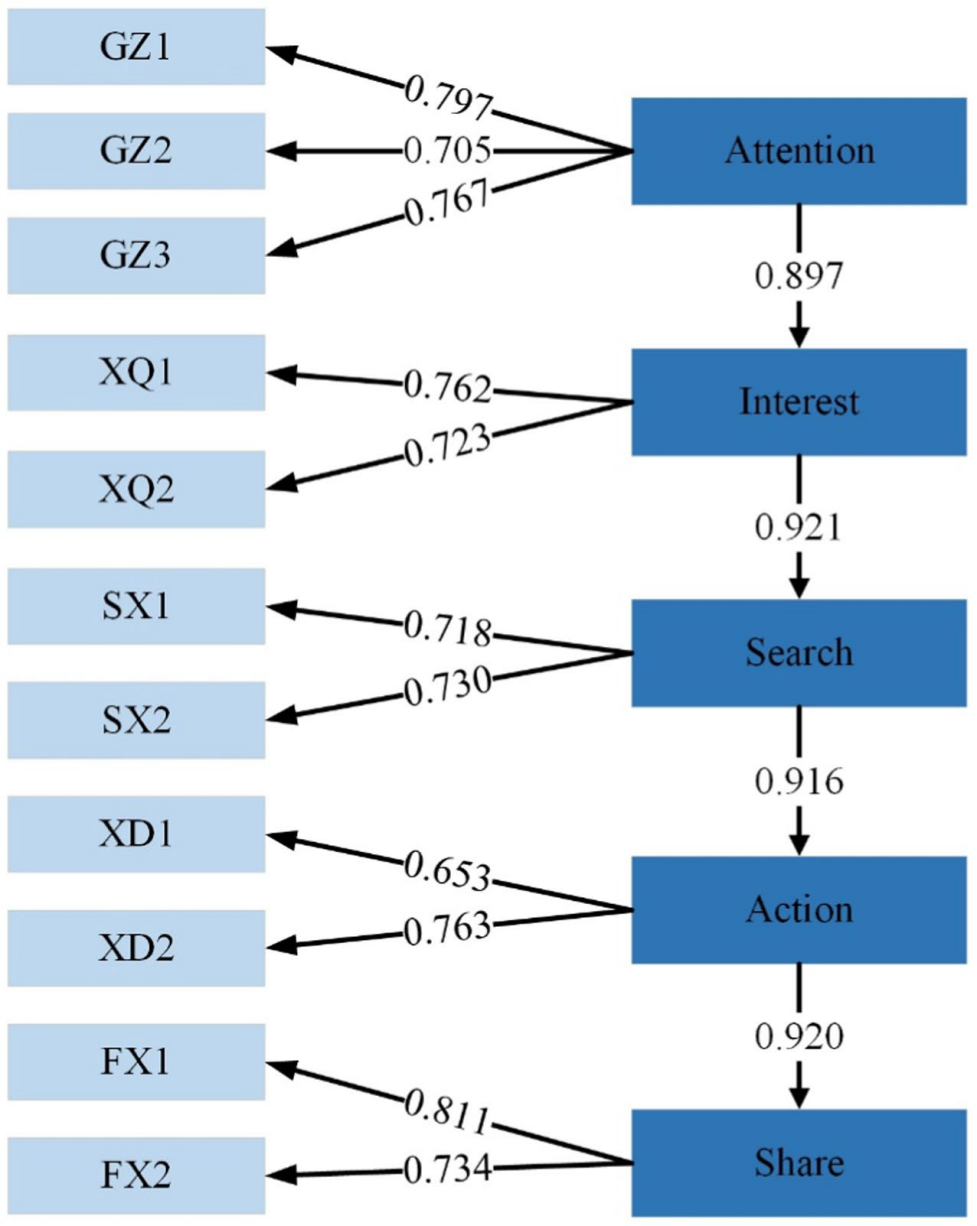
SEM model result graph.

The structural equation model (SEM) visually presents the correlation between variables in the form of a path graph. The SEM can observe variables (IP content, IP value, emotional trust, spiritual consumption, fan interaction) and judge the quality of the model. This article analyzes the questionnaire data by importing it into AMOS20.0 software, and the resulting model is shown in Figure 3.

Based on the research, this paper selected several of the most representative detection indicators to conduct a comprehensive assessment of the measurement model’s goodness of fit. The model fit indicators are presented in Table 5, in which CMIN/DF refers to the chi-square/degree of freedom, the smaller the value means the better the model fit. CFI is the comparative fit index, the closer the value is to 1, the better the fit, NFI is the normative fit index, the closer the value is to 1, the better the fit, NNFI is the non-standardized fit index, the closer the value is to 1, the better the fit.

**Table 5:** Model fitting results.

Index name	CMIN/DF	GFI	RMSEA	RMR	CFI	NFI	NNFI
Standard	<3	>0.9	<0.05	<0.05	>0.9	>0.9	>0.9
Measured value	1.750	0.940	0.059	0.036	0.975	0.944	0.966

In our structural equation modeling (SEM) analysis, we first focused on the Chi-Square/Degrees of Freedom ratio (CMIN/DF). This ratio is used to assess the overall fit of the model. The standard is less than 3, and our model scored 1.750 in this index, well below this threshold. This result indicates that our model fits the data very well, suggesting that the model structure is appropriate. Next, we examined the Goodness of Fit Index (GFI). This index reflects the degree to which the model fits the sample data. The ideal GFI value should be over 0.9, and our model scored 0.940 in this index. This indicates that our model can accurately reflect the observed data patterns, proving the appropriateness of the model design. The Root Mean Square Error of Approximation (RMSEA) is another important indicator, used to measure the approximate error of the model. Although the ideal RMSEA value is less than 0.05, our model scored 0.059, slightly exceeding the ideal range. Nevertheless, this value is still close to the ideal standard and can be considered an acceptable level of fit, indicating that the model is overall appropriate. We also evaluated the Standardized Root Mean Square Residual (RMR). The lower this index, the smaller the residuals of the model, indicating a better fit. Our model scored 0.036 in RMR, well below the standard of 0.05, meaning that the residuals of the model are very small, and the fit is good. The Comparative Fit Index (CFI), Normed Fit Index (NFI), and Non-Normed Fit Index (NNFI), also known as TLI, are three other key indicators for assessing the fit of the model. The ideal values for these indices are all above 0.9. In our model, CFI was 0.975, NFI was 0.944, and NNFI was 0.966. These results are significantly higher than the 0.9 standard, indicating that our model demonstrates excellent fit in these indices. In conclusion, our structural equation model shows good or excellent performance in several key fit indices. Although the RMSEA is slightly above the ideal value, it is still within an acceptable range, indicating that our model is overall appropriate and effective.

Table 6 mainly shows the results of model regression coefficient analysis, if *p* < 0.001, it explain the path relationship is significant. S.E.: Measures load estimate uncertainty, smaller values mean more reliability. C.R.: Tests if load estimates significantly differ from zero, larger values imply significance. *P*: value of *p*, shows probability of significant load estimates (usually <0.05). CR: Measures internal consistency of latent variables (higher values indicate reliability). AVE: Reflects convergent validity, should be >0.5 for good validity. According to the actual results of this table, we can draw the following conclusions: (1) Transitioning from the attention to interest phase, we found a highly significant positive impact, as evidenced by the high NSRC (0.861) and nearly perfect SRC (0.965). The CR value of 10.897 and a value of *p* of 0.000 further validate the strong influence of the attention phase on the interest phase. (2) In the transition from interest to search, we observed a very strong positive relationship. The NSRC value is 1.031, and the standardized regression coefficient reached 1.000, indicating the significance of the interest phase in impacting the search phase. The CR value of 10.423 and a value of *p* of 0.000 demonstrate the statistical significance of this relationship. (3) In the link from search to action, we noted a significant positive influence, reflected in an NSRC of 0.872 and SRC of 0.925. The CR value of 8.579 and a value of *p* of 0.000 once again emphasize the pivotal role of the search phase in prompting the action phase. (4) The influence of the action phase on the share phase is also significant, with an NSRC of 1.123 and an SRC of 0.943 indicating this strong positive impact. The CR value of 9.294 and a value of *p* of 0.000 indicate the significant role of the action phase in driving the sharing process. (5) We also found that the attention, interest, search, action, and share phases have significant positive relationships with their corresponding measurement items. The high standardized regression coefficients in these relationships indicate that not only do the phases of the model interact with each other, but they are also closely related to their measurement items.

**Table 6:** Model regression coefficient analysis.

*X*	→	*Y*	NSRC	SE	*z* (CR)	*p*	SRC
Attention	→	Interest	0.861	0.079	10.897	0.000	0.965
Interest	→	Search	1.031	0.099	10.423	0.000	1.000
Search	→	Action	0.872	0.102	8.579	0.000	0.925
Action	→	Share	1.123	0.121	9.294	0.000	0.943
Attention	→	GZ1	1.000	–	–	–	0.797
	→	GZ2	0.895	0.083	10.756	0.000	0.705
	→	GZ3	0.998	0.084	11.916	0.000	0.767
Interest	→	XQ1	1.000	–	–	–	0.762
	→	XQ2	1.066	0.099	10.751	0.000	0.723
Search	→	SX1	1.000	–	–	–	0.718
	→	SX2	1.065	0.104	10.215	0.000	0.730
Action	→	XD1	1.000	–	–	–	0.653
	→	XD2	1.134	0.121	9.339	0.000	0.763
Share	→	FX1	1.000	–	–	–	0.811
	→	FX2	0.975	0.090	10.784	0.000	0.734

→ Indicates regression influence relationship or measurement relationship. NSRC, non-standardized regression coefficient; SE, standard error; CR, critical ratio; SRC, standardized regression coefficient; *p*, value of *p*, indicating the level of statistical significance of the test statistic under the null hypothesis. Typically, a value of *p* of less than 0.05 is considered statistically significant.

Overall, our analysis shows that each phase of the model has a significant positive effect on the subsequent phase, and these phases are also closely related to their measurement items. The high CR values and very low value of *p*s statistically confirm the importance of these findings.

## 5 Discussion

This study reveals the important role of IP content in shaping consumer behavior through an in-depth analysis of structural equation modeling (SEM). Key findings support our hypotheses about the role of IP content, value, emotional trust, spiritual consumption, and fan interaction in the consumer decision-making process.

The significant positive impact of IP content (H1) on the consumer attention stage reveals a key insight: in IP marketing, consumers are initially attracted to the unique image and creative content of the IP. This attraction is not only key to marketing success, but also the basis for building a long-term relationship between the consumer and the brand. Therefore, brands need to invest resources in creating and maintaining engaging IP content as an effective means of attracting and keeping consumer attention.

When discussing IP value (H2), we found that cultural and emotional values play a crucial role in shaping consumer identity and interest. Consumers often look for brands that match their values and cultural backgrounds, so IP needs to embody cultural and emotional qualities that resonate with the target consumer group. This resonance not only enhances brand loyalty, but also drives word-of-mouth and social sharing.

The positive role of emotional trust (H3) in the consumer search phase emphasizes the importance of brand trust in e-commerce and virtual interactions. Consumers, in the absence of a physical experience, are more inclined to trust IPs that provide consistent and reliable information. Therefore, maintaining transparency and consistency, as well as providing high-quality content, is critical to building and maintaining consumer trust.

The positive impact of Spiritual Consumption (H4) highlights the trend for consumers to place increasing emphasis on personal value and emotional fulfillment in their purchasing decisions. In today’s consumer culture, consumers are not only buying products, they are seeking experiences that align with their personal emotions and values. Therefore, brands need to create stories and experiences that resonate with consumers’ emotions through IP to promote deeper consumer engagement.

The positive effect of fan interaction (H5) on the sharing phase proves the importance of social interaction in enhancing the relationship between consumers and brands. Through effective social media strategies and fan interactions, brands can not only increase the visibility and engagement of their content, but also expand their reach through their fans’ social networks.

Furthermore, our analyses show that factors at each stage play an important role in the consumer decision-making process and have a significant influence on each other. This emphasizes the importance of considering the entire consumer decision-making process in a brand’s strategy to ensure effective engagement and communication with consumers at every stage.

## 6 Conclusion

This study explores the role of IP content in influencing consumer behavior through SEM analysis, revealing the importance of IP content, value, emotional trust, spiritual consumption and fan interaction. While our findings provide new insights into the influence of IP marketing, there are some limitations. In particular, our study focuses on specific markets and IP types and may not be broadly applicable to all contexts. The contribution of this study is to establish a theoretical basis for understanding how IP content influences consumer decisions. This provides brands with strategies to utilize IP content for effective marketing, particularly in shaping consumers’ emotional connection to IP.

Looking towards future research, we suggest further exploring the application of the AISAS model in the area of emotional marketing. In particular, it is important to investigate how emotions play a role in attracting consumers’ attention, sparking interest, guiding searches, facilitating purchasing behaviors, and sharing behaviors. For example, how affective strategies influence consumer attraction during the attention phase or how positive emotions are maintained and enhanced during the interest phase. It is also worth exploring how emotional marketing content plays a role in search and purchase decisions, as well as how it motivates consumers to share experiences and recommend products.

Another important direction is to analyze the difference between ‘interest’ and ‘desire’ in consumer behavior. Interest may be based more on rational and conscious evaluation, whereas desire is closely related to emotionally driven and subjective impulses. This difference has a profound impact on consumers’ purchasing decisions and brand loyalty. For example, when analyzing a specific product, it is possible to explore how consumers move from an interest in the product to a strong desire for it, and how this shift affects their purchasing behavior.

In conclusion, this study highlights the importance of understanding and using IP content in modern marketing strategies and opens up new avenues for future research in the field of emotional marketing and consumer psychoanalysis.

## Data availability statement

The raw data supporting the conclusions of this article will be made available by the authors, without undue reservation.

## Ethics statement

Ethical review and approval was not required for the study on human participants in accordance with the local legislation and institutional requirements. Written informed consent from the [patients/participants OR patients/participants legal guardian/next of kin] was not required to participate in this study in accordance with the national legislation and the institutional requirements.

## Author contributions

HZ: Conceptualization, Data curation, Formal analysis, Investigation, Project administration, Software, Writing – original draft, Writing – review & editing. XL: Funding acquisition, Resources, Supervision, Validation, Visualization, Writing – review & editing.
